# Persistence of Gut Microbiota Dysbiosis and Chronic Systemic Inflammation After Cerebral Infarction in Cynomolgus Monkeys

**DOI:** 10.3389/fneur.2019.00661

**Published:** 2019-06-28

**Authors:** Yonghong Chen, Jiahui Liang, Fubing Ouyang, Xinran Chen, Tao Lu, Zimu Jiang, Jianle Li, Yuefeng Li, Jinsheng Zeng

**Affiliations:** ^1^Department of Neurology and Stroke Center, The First Affiliated Hospital, Sun Yat-sen University, Guangzhou, China; ^2^Department of Neurology, Liuzhou Worker's Hospital, Fourth Affiliated Hospital of Guangxi Medical University, Liuzhou, China; ^3^Guangdong Landau Biotechnology Co., Ltd., Guangzhou, China

**Keywords:** gut microbiota, short-chain fatty acids, intestinal mucosal damage, systemic inflammation, cerebral infarction, middle cerebral artery occlusion, cynomolgus monkey

## Abstract

**Background:** The bidirectional interaction between the gut and brain after stroke through the immune-mediated pathway has been studied. However, the long-term effects of gut microbiota and systemic immune homeostasis after cerebral ischemia remain unclear. We examined long-term changes in the gut microbiota and systemic inflammatory cytokines after cerebral infarction in cynomolgus monkeys.

**Methods:** Twelve monkeys underwent successful distal M1 segment of the left middle cerebral artery occlusion (MCAO) and were randomly and equally assigned to the MCAO-1.5 m, MCAO-6 m, and MCAO-12 m groups, which were sacrificed 1.5, 6, and 12 months after cerebral infarction induction, respectively. Four monkeys that underwent a sham operation were sacrificed 12 months later. The gut microbiota and short-chain fatty acids (SCFAs) were analyzed by 16S rDNA sequencing and gas chromatography mass spectrometry, respectively. Histological examinations of the transverse colon were performed. Plasma D-lactate, zonulin, lipopolysaccharide (LPS), tumor necrosis factor (TNF-α), interferon (IFN)-γ, and interleukin (IL)-6 were detected by immunoassay kits.

**Results:** The levels of the Bacteroidetes phylum and *Prevotella* genus were significantly increased, while the Firmicutes phylum as well as the *Faecalibacterium, Oscillospira*, and *Lactobacillus* genera were decreased after cerebral infarction. Gut-originating SCFAs were significantly decreased 6 and 12 months after cerebral infarction (*P* < 0.05). We observed intestinal mucosal damage, evaluated by Chiu's score. Plasma D-lactate, zonulin, LPS, TNF-α, IFN-γ, and IL-6 were significantly increased after cerebral infarction (*P* < 0.05). Additionally, the increases in plasma LPS, TNF-α, IFN-γ, and IL-6 after cerebral infarction coincided with overgrowth of the Bacteroidetes phylum (*P* < 0.001).

**Conclusion:** Cerebral infarction induces persistent host gut microbiota dysbiosis, intestinal mucosal damage, and chronic systemic inflammation in cynomolgus monkeys.

## Introduction

The gut–brain axis plays an important role in the bidirectional communication between the gut and brain (1, 2). Emerging evidence suggests that gut microbiota dysbiosis can exert immunomodulatory effects on the progression and development of neurological diseases, such as stroke (3, 4). However, most studies investigating the interaction of the gut microbiota with the brain and immune system are based on rodent findings, and rodents are significantly different from humans in terms of neuroanatomy, the gastrointestinal tract, and behavior (e.g., coprophagia), which may influence the gut microbiota (5, 6). Thus, rodent studies are limited with regard to poststroke gut microbiota dysbiosis in humans. Gut microbiota dysbiosis has also been shown in ischemic stroke patients (7). Nevertheless, clinical studies on the gut microbiota in stroke patients also have limitations. Interindividual variation in daily diet (8) as well as comorbidities such as hypertension (9), diabetes (10), and obesity (11) may influence the gut microbiota in stroke patients. Non-human primates (NHPs) share more similarities with humans regarding immunology, genetic structure, feeding behavior, neuroanatomy, and gastrointestinal tract structure than do rodents (6, 12, 13) and are therefore an excellent model for studying the influence of the poststroke brain–gut microbiota axis.

Gut microbiota dysbiosis can disrupt systemic immune homeostasis by gut-originating metabolic products such as short-chain fatty acids (SCFAs) and proinflammatory factors involved in the gut–brain axis (14–16). SCFAs (acetate, propionate, and butyrate) are the main metabolic products of the gut microbiota, especially in the intestine (14). They are energy sources for intestinal epithelial cells and are also crucial for gut immune homeostasis (17). Lipopolysaccharide (LPS), a potent proinflammatory factor originating from gram-negative bacteria, can induce systemic inflammation, especially when the intestinal mucosal barrier is disrupted (18). In a mouse model, gut microbiota dysbiosis after stroke had been demonstrated affecting the stroke outcome through the immune-mediated pathway (3, 4). However, since the experimental observation time was within 1 week, the long-term effects of cerebral ischemia on the gut microbiota or systemic inflammation were not evaluated.

In this study, we aimed to provide evidence characterizing the long-term changes in gut microbiota and systemic immune homeostasis using a cynomolgus monkey cerebral infarction model. We tested whether a disturbance in the gut microbiota composition was accompanied by a reduction in SCFAs and whether the intestinal mucosa was damaged after cerebral infarction. Furthermore, the permeability of the intestinal mucosal barrier was assessed by measuring D-lactate, zonulin, and LPS levels in plasma. The inflammatory cytokines tumor necrosis factor (TNF)-α, interferon (IFN)-γ, and interleukin (IL)-6 in the plasma were also detected.

## Materials and Methods

### Animals Preparation and Ethics Approval

Twenty-three male cynomolgus monkeys (aged 4–5 years, weighing 5.5–6 kg) were obtained from Guangdong Landau Biotechnology Co., Ltd. (Guangzhou, China), which is an Association for Assessment and Accreditation of Laboratory Animal Care-accredited facility. All monkeys were housed individually under a constant temperature (24–28°C), humidity (55–65%), and light–dark cycle (12:12 h). All monkeys had *ad libitum* access to drinking water and were fed twice daily with monkey chow (Guangzhou Feed Research Institute, Guangzhou, China) supplemented with fruits and vegetables disinfected by ultraviolet treatment. The cages were sterilized with 75% ethanol, and excrement was cleaned up twice a day. All experimental procedures were performed in accordance with the Animal Research: Reporting of *in vivo* Experiments (ARRIVE) guidelines for the care and use of laboratory animals and were approved by the Institutional Animal Care and Use Committee of Guangdong Landau Biotechnology Co., Ltd. (IACUC, Approval No: LDS2017-001). The health status of all monkeys was monitored during this study.

### Middle Cerebral Artery Occlusion and Sham Operation

Cerebral infarction was induced in monkeys by distal M1 segment of the left middle cerebral artery occlusion (MCAO) as described in our previous study (19). In brief, ketamine (10 mg/kg) was administered intramuscularly for anesthesia induction. Then, a tracheal cannula was inserted, and 1% isoflurane mixed with oxygen was administered *via* inhalation for anesthetic maintenance. Blood oxygen saturation, respiration, and the electrocardiogram were monitored during the operation. Then, an incision was made on the left side of the temporal scalp. Skull and dura mater on the pterion were removed to expose the sylvian fissure. Then, the distal M1 segment of the middle cerebral artery was exposed carefully, and bipolar electrocoagulation was performed for permanent occlusion of the artery. Finally, the scalp was sutured, and the tracheal cannula was removed. The procedure for the sham operation was similar to that for the MCAO surgery, the distal M1 segment of the left middle cerebral artery (MCA) was exposed but without bipolar electrocoagulation. After the operation, all the monkeys were administered with penicillin (0.4 million IU) by intramuscular injection once a day for 2 days, and the wound was treated with skin disinfectant for 1 week to prevent postoperative infection. Tramadol (4 mg/kg) was intramuscularly administered once a day for 2 days to ease pain. Within 1 week after the operation, all monkeys were housed in home cages at a constant temperature. The cages were sterilized with 75% ethanol, and the excrement was cleaned up twice a day. The food consumption, excrement feature, consciousness, responses to stimulation, posture, appearance, and movements of the monkeys were continuously monitored, and intensive care was provided by experienced veterinarians. All monkeys were fed with additional rice paste within 7 days after the operation. The wound healed within 7 to 10 days. No signs of infection were observed during our study.

Twelve monkeys successfully underwent MCAO and were randomly and equally assigned to the MCAO-1.5 m, MCAO-6 m, and MCAO-12 m groups (*n* = 4 per group), which were sacrificed 1.5 months, 6 months, and 12 months after cerebral infarction induction, respectively. Four monkeys that underwent the sham operation were sacrificed 12 months later (sham-12 m, *n* = 4). To confirm whether penicillin administration affected the gut microbiota in our study, six monkeys were randomly and equally assigned to receive an intramuscular injection of 0.4 million IU penicillin (antibiotic group, *n* = 3) or saline (vehicle group, *n* = 3) once a day for 2 days. One monkey was excluded because of MCAO failure.

### Magnetic Resonance Imaging (MRI) Acquisition and Analysis

MRI scans were performed before the operation and then 7 days and 1 month later on a Siemens 3.0-Tesla Verio scanner (Siemens, Erlangen, Germany) with an 8-channel monkey head coil fixed on a stereotaxic instrument (Suzhou Medcoil Healthcare Co., Ltd, Suzhou, China). The monkeys were anesthetized with ketamine (10 mg/kg, intramuscular injection) and pentobarbital (8 mg/kg, intravenous injection), and their vital signs were continuously monitored. The acquisition parameters were as follows: T2-weighted images [repetition time (TR) = 4,330 ms, echo time (TE) = 105 ms, matrix = 320 × 320, field of view (FOV) = 120 mm × 120 mm, and flip angle = 150°], magnetic resonance angiography (MRA) (TR = 21.4 ms, TE = 3.6 ms, FOV = 150 mm × 150 mm, and flip angle = 18°), and T1-weighted magnetic prepared rapid acquisition gradient echo (T1-MPRAGE) (TR = 2,000 ms, TE = 3.4 ms, inversion time = 800 ms, matrix = 256 × 256, FOV = 150 mm × 150 mm, and flip angle = 12°).

Two experienced researchers independently analyzed coronal slices of T2-weighted images and maximum intensity projections of MRA images to confirm the permanent occlusion of the distal M1 segment on the left MCA and infarction region 7 days after the MCAO operation. The cerebral infarction volume for each monkey was measured on T1-MPRAGE images obtained 1 month after MCAO. In brief, the infarct lesion and ipsilateral hemisphere (region of interest) were manually drawn and measured by ITK-SNAP (20). The infarct volume proportion was calculated by the following formula: (infarct volume/ipsilateral hemisphere volume) × 100%.

### Neurological Function Assessment

Neurological function assessments were performed before and 1 week after the operation by two researchers blinded to the grouping data using a standardized neurological deficit scale to evaluate consciousness, motor function, sensory function, and muscular coordination (21). A score of 0 indicates normal neurological function, while a total score of 100 indicates the severe bilateral neurological function impairment.

### Fecal Sample Collection and Microbial Community Analysis

Fecal samples from MCAO and sham-operated monkeys were collected 1 day before sacrifice. Briefly, the home cages were sterilized using 75% ethanol. The activities of the monkeys were continuously monitored. When a monkey was defecating, a sterilized container was immediately placed under the home cage to collect fecal samples. Fecal samples contaminated by fur or urine were excluded and recollection was performed. For monkeys in the antibiotic and vehicle groups, fecal samples were collected 1.5 months after antibiotic or vehicle administration. Fresh feces were immediately frozen and stored at −80°C after collection. All fecal samples were analyzed in one batch in our study.

DNA from the fecal samples was isolated using a TIANamp stool DNA Kit (Tiangen, Beijing, China, Cat #DP328) according to the manufacturer's instructions. 16S rDNA was amplified using primers corresponding to the V4 regions (515F: GTGCCAGCMGCCGCGGTAA; and 806R: GGACTACHVGGGTWTCTAAT), and the polymerase chain reaction (PCR) cycling conditions were as follows: initial denaturation at 98°C for 30 s, 35 cycles at 98°C for 10 s, 54°C for 30 s, and 72°C for 45 s, and a final extension step at 72°C for 10 min. The PCR products were purified and sequenced on an Illumina MiSeq PE250 according to the manufacturer's instructions.

The raw sequences were preprocessed according to the barcoded Illumina paired-end sequencing (BIPES) workflow (22), and quality filtering was performed on the raw tags under specific filtering conditions to obtain high-quality clean tags according to fqtrim (v0.94). USEARCH (v7.0.1090) was used to filter chimeric sequences and assign sequences with ≥97% similarity to the same operational taxonomic units (OTUs) (23). Taxonomic data were then assigned to each representative sequence of each OTU using the RDP (Ribosomal Database Project) classifier. OTU abundance information was normalized by a standard sequence number corresponding to the sample with the fewest sequences. Alpha diversity represents the richness and evenness of within-sample microbiota species diversity, which can be measured by the Shannon index (24). A higher Shannon index indicates greater within-sample microbiota species diversity (24). Furthermore, beta diversity represents between-sample microbiota species diversity (24). The weighted UniFrac distance represents both species counts and richness between each sample and is an important marker of beta diversity (25). Microbiota dysbiosis was measured by the relative abundance of each taxonomy in the microbiome at the phylum and genus levels. The above indices were analyzed according to QIIME (v1.9.1) (26). To quantitatively determine significant differences in the microbiota, the linear discriminant analysis effect size (LEfSe) (27) was calculated to compare the MCAO-1.5 m, MCAO-6 m, and MCAO-12 m groups with the sham-12 m group. A higher absolute value for the LDA score indicates a greater difference in a specific microbial taxon between groups. The alpha level was set to 0.05. The threshold for the linear discriminant analysis (LDA) score was 3.5, and the LDA score was used to evaluate differences in the microbiota between the MCAO groups and the sham-12 m group.

### Fecal SCFA Analysis

The SCFAs in the fecal samples were measured using an Agilent 7890A gas chromatography system coupled to an Agilent 5975C inert XL EI/CI mass spectrometric detector (Agilent, San Diego, USA) as previously described (28). A volume of 1 ml of 0.005 M NaOH was added to 100 mg of fecal sample. The mixture was homogenized for 10 min and centrifuged at 13,200 × *g* at 4°C for 20 min. The supernatant was extracted and derivatized with a PrOH/pyridine solution (3:2, v/v) and propyl chloroformate, and the derivatives were further extracted by hexane. The extracts of the SCFAs were loaded in a polar DB-WAX capillary column (30 m × 0.25 mm i.d., 0.25-μm film thickness, Agilent, CA). Helium was used as a carrier gas and pumped at a constant flow rate of 1 ml/min. Initially, the temperature in the oven was maintained at 60°C for 5 min, elevated to 250°C at a rate of 10°C/min, and finally maintained at this temperature for 5 min. The temperatures of the front inlet, transfer line, and electron impact ion source were set at 280°C, 250°C, and 230°C, respectively. Data analysis was performed on an Agilent's MSD ChemStation (E.02.00.493, Agilent Technologies, Inc., USA).

### Evaluation of Intestinal Mucosal Histopathology

Before sacrifice, the monkeys were fasted for solids overnight. The monkeys were anesthetized with ketamine (10 mg/kg, intramuscular injection) and an overdose of pentobarbital (20 mg/kg, intravenous injection) to induce deep anesthesia. Then, the monkeys were transcardially perfused with ice-cold saline and 4% paraformaldehyde solution. Transverse colon sections were removed and postfixed in 4% paraformaldehyde solution. A portion of each transverse colon section was paraffin-embedded, and 4-μm-thick slices were cut for further analysis. The slices were stained with a hematoxylin and eosin (HE) staining kit (Baso, Zhuhai, China Cat #BA4025) according to the manufacturer's instructions. The histopathological assessment was performed by light microscopy at 200 × magnification to identify morphological changes in the intestinal mucosa according to Chiu et al. (29). The severity of injury was graded from 0 to 5 as follows: Grade 0, normal mucosal villi; grade 1, mild development of subepithelial Gruenhagen's spaces; grade 2, extension of the subepithelial space with moderate progressive lifting of the epithelial layer from the lamina propria; grade 3, severe epithelial lifting down the sides of villi; grade 4, completely denuded villi with exposed lamina propria and dilated capillaries; and grade 5, disintegration of the lamina propria, hemorrhage, and ulceration. The extent of mucosal damage was evaluated by two pathologists blinded to this study using a minimum of three randomly selected fields from each sample.

### Immunoassay

Blood samples were collected 1 day before the MCAO and sham-operated monkeys were sacrificed. Ethylenediaminetetraacetic acid (EDTA) was used for anticoagulation in the blood samples, which were centrifuged at 3,500 rpm for 5 min at 4°C. Then, fresh plasma was immediately frozen and stored at −80°C. All plasma was analyzed in one batch in our study. The plasma was then extracted for enzyme-linked immunosorbent assay (ELISA). Commercial ELISA kits were used to detect the levels of D-lactate (BioVision, Milpitas, USA, Cat #E4356-100), zonulin (Cusabio, Wuhan, China, Cat #CSB-EQ027649HU), and LPS (USCN Life Science, Wuhan, China, Cat #E1526Ge), which are biomarkers of increased intestinal permeability (18, 30, 31). Moreover, the inflammatory cytokines TNF-α, IFN-γ, and IL-6 were also detected in plasma by immunoassay kit (Millipore, Billerica, USA, Cat #HSTCMAG-28SK). All kits were used according to the manufacturers' instructions.

### Statistical Analysis

All experimental data were analyzed using SPSS v23.0 software for Windows (IBM, New York, USA) and are shown as the mean ± SD for Gaussian distributed data or the median and interquartile for non-Gaussian distributed data. One-way ANOVA and the Kruskal–Wallis test were used to analyze Gaussian distributed data and non-Gaussian distributed data, respectively. Each MCAO-operated group was compared with the sham-12 m group, and Dunn's multiple comparison test was used for the *post hoc* analysis. Correlations between the Bacteroidetes level and LPS, TNF-α, IFN-γ, and IL-6 levels were analyzed by Spearman correlation analysis. The level of statistical significance was set to 0.05.

## Results

### Identification of Vessel Occlusion and the Infarction Region After Surgery

**Figure 1:**
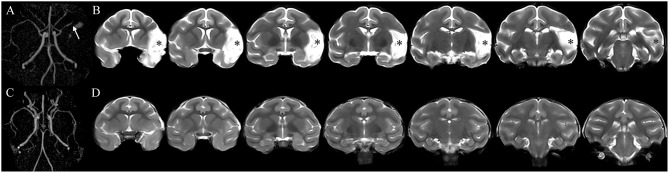
The occluded vessel and initial infarct were confirmed by magnetic resonance angiography (MRA) and T2-weighted images obtained 1 month after surgery. **(A)** The monkey underwent cerebral infarction induction by permanent occlusion of the distal M1 segment of the left middle cerebral artery (MCA). The arrowhead shows the occlusion of the left MCA. **(B)** The asterisk indicates the left infarct lesion. **(C,D)** The monkey received a sham operation without occlusion of the left MCA and infarct lesion.

All monkeys in the MCAO groups exhibited permanent occlusion of the distal M1 segment of the left MCA on MRA images obtained 1 month after the operation (Figure 1A). A hyperintensive infarct lesion was detected on the coronal T2-weighted images (Figure 1B). The mean infarct volume of the monkeys in the MCAO groups 1 month after the operation was 15.5 ± 2.4% (Supplementary Figure 1). No statistical difference in the mean infarct volume was found among all MCAO groups (Supplementary Figure 1). The sham-operated monkeys displayed neither MCA occlusion nor cerebral infarcts on MRA or coronal T2-weighted images obtained 1 month after the operation (Figures 1C,D).

### The MCAO Operation-Induced Neurological Deficits

In general, after the MCAO operation, the monkeys presented palsy and hypoalgesia of the right upper limb, incoordination of the skeletal muscles, and mild to moderate drowsiness. The sham-operated monkeys were conscious but clouded and fully recovered within 2 days. The standardized neurological deficit scores 1 week after the operation in the MCAO-1.5 m, MCAO-6 m, and MCAO-12 m groups were 38.25 ± 1.50, 37.75 ± 2.99, and 37.25 ± 3.30, respectively. No statistical difference was found between the MCAO groups (*P* > 0.05). In addition, the sham operation did not induce neurological deficits among the monkeys in the sham-12 m group. The standardized neurological deficit score 1 week after the operation in the sham-12 m group was 0.

### Gut Microbiota Alterations After MCAO

**Figure 2:**
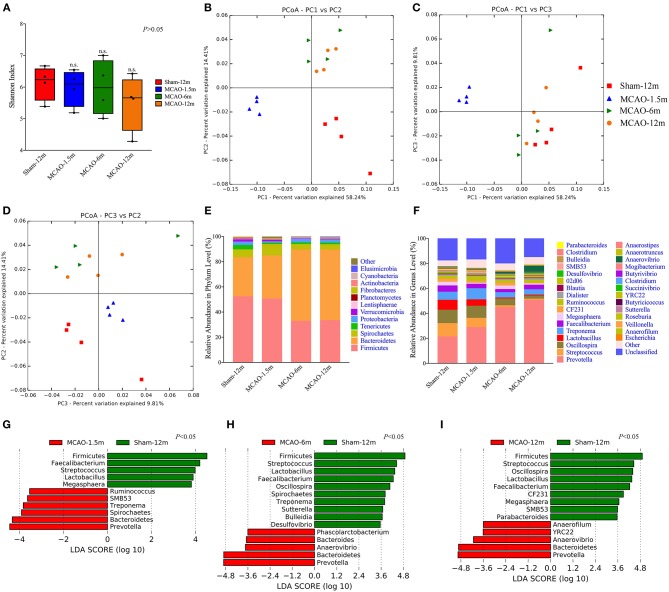
Comparison of the gut microbiota between the sham-operated monkeys and permanent middle cerebral artery occlusion (MCAO)-operated monkeys. **(A)** Shannon indices showed no significant differences in alpha diversity among the sham-12 m (6.235, 5.574–6.586), MCAO-1.5 m (6.095, 5.375–6.473), MCAO-6 m (5.980, 5.153–6.843), and MCAO-12 m (5.661, 4.621–6.241) groups (*P* > 0.05). **(B–D)** Principal coordinate analysis (PCoA) of the intestinal microbiota was performed by the weighted UniFrac distance. **(E)** Taxonomic summary of the microbiota at the phylum level. **(F)** Taxonomic summary of the microbiota at the genus level. **(G–I)** The linear discriminant analysis effect size (LEfSe) showed increased relative abundance levels of the Bacteroidetes phylum (*P* < 0.05) and *Prevotella* genus (which belongs to Bacteroidetes) (*P* < 0.05) and decreased relative abundance of the Firmicutes phylum (*P* < 0.05) and *Faecalibacterium, Streptococcus*, and *Lactobacillus* genera (which belong to Firmicutes) (*P* < 0.05) in all MCAO groups. In addition, a decreased relative abundance of the *Oscillospira* genus (which belongs to Firmicutes) was found in the MCAO-6 m and MCAO-12 m groups (*P* < 0.05).

In our study, the alpha diversity (represented by the Shannon index) of each sample was analyzed to evaluate within-sample species diversity. The within-sample species diversity between the MCAO and sham-operated monkeys did not significantly differ (*P* > 0.05; Figure 2A).

Beta diversity indicates the between-sample species diversity and reflects differences in the gut microbiota between the MCAO groups and sham-12 m group in our study. The weighted Unifrac distance was used to measure beta diversity between the MCAO groups and sham-12 m group. PCoA plotted by using the weighted Unifrac distance showed clear separate grouping patterns, which indicated that the richness and counts of the gut microbiome communities in the sham-12 m group differed from those in the MCAO groups (Figures 2B–D). On the other hand, the results of the monkeys in the MCAO-6 m and MCAO-12 m groups were grouped together, indicating that the monkeys in the MCAO-6 m and MCAO-12 m groups had similar richness and counts of the gut microbiota (Figures 2B–D). In conclusion, the gut microbiota in the monkeys in the MCAO groups showed significant differences from that in the sham-12 m group.

Firmicutes and Bacteroidetes were the dominant phyla in all groups (Figure 2E). In addition, the *Prevotella, Faecalibacterium, Streptococcus, Lactobacillus*, and *Oscillospira* genera constituted the majority of the gut microbiota in all groups (Figure 2F). Furthermore, we applied the LEfSe to quantitatively investigate differences in the gut microbiota between the MCAO groups and sham-12 m group. In terms of alterations of the major gut microbiome communities, compared with the sham-12 m group, all MCAO groups exhibited increases in the relative abundance levels of the Bacteroidetes phylum and *Prevotella* genus and decreases in the relative abundance levels of the Firmicutes phylum and *Faecalibacterium, Streptococcus*, and *Lactobacillus* genera (*P* < 0.05; Figures 2G–I). In addition, a decreased relative abundance of the *Oscillospira* genus was found in the MCAO-6 m and MCAO-12 m groups (*P* < 0.05; Figures 2H,I). Furthermore, the abundance of the minor gut microbiome genera such as the Spirochaetes phylum and *Treponema, SMB53, Ruminococcus, Megasphaera, Sutterella, Desulfovibrio, Phascolarctobacterium, Anaerovibrio, CF231, Parabacteroides, Anaerofilum*, and *YRC22* genera was also different among the MCAO groups and the sham-12 m group (*P* < 0.05; Figures 2G–I). The changes of gut microbiota at the family level were also provided in supplementary materials (Supplementary Figure 3).

In our study, penicillin was used to prevent post-surgery infection. To investigate the impact of penicillin on the gut microbiota, we compared the relative abundance levels of the gut microbiota in the antibiotic group with those in the vehicle group 1.5 months after intramuscular penicillin injection (Supplementary Figure 2). LEfSe analysis indicated no significant difference between the antibiotic and vehicle groups. Penicillin usage in our study had no effect on the gut microbiota.

### Reduction in SCFAs After MCAO

**Figure 3:**
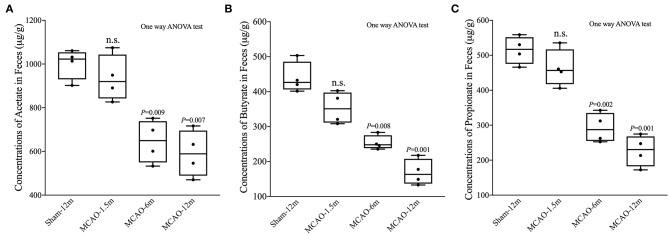
Production of short-chain fatty acid (SCFA; acetate, propionate, and butyrate) between the sham-operated monkeys and permanent middle cerebral artery occlusion (MCAO)-operated monkeys. **(A)** No significant differences in acetate were found between the MCAO-1.5 m (935.1 ± 105.5 μg/g) and sham-12 m (1,002.0 ± 69.4 μg/g) groups, while the concentrations of acetate in the MCAO-6 m (645.6 ± 97.8 μg/g) and MCAO-12 m (591.2 ± 106.5 μg/g) groups were significantly decreased compared with that in the sham-12 m group. **(B)** The concentration of butyrate was significantly lower in the MCAO-6 m (253.7 ± 20.7 μg/g) and MCAO-12 m (169.3 ± 37.2 μg/g) groups than that in the sham-12 m group (439.5 ± 44.5 μg/g). No significant differences in the concentration of butyrate were observed in the MCAO-1.5 m group (353.2 ± 45.8 μg/g) compared with that in the sham-12 m group. **(C)** The propionate concentration was significantly lower in the MCAO-6 m (292.4 ± 42.5 μg/g) and MCAO-12 m (226.9 ± 44.2 μg/g) groups than that in the sham-12 m group (439.5 ± 44.5 μg/g). No significant difference in the propionate concentration was observed between the MCAO-1.5 m (514.7 ± 39.5 μg/g) group and the sham-12 m group (514.7 ± 39.5 μg/g). The data are expressed as the mean ± SD; *n* = 4 monkeys per group.

We also detected SCFA (acetate, propionate, and butyrate) levels in fecal samples. The concentrations of acetate were not statistically different between the MCAO-1.5 m group and the sham-12 m group (*P* > 0.05; Figure 3A), whereas the concentrations of acetate in the MCAO-6 m and MCAO-12 m groups were lower than that in the sham-12 m group (*P* < 0.05; Figure 3A). The concentrations of butyrate and propionate were lower in the MCAO-6 m and MCAO-12 m groups than those in the sham-12 m group (*P* < 0.01; Figures 3B,C), but no statistical differences in the concentrations of propionate or butyrate were observed between the MCAO-1.5 m group and the sham-12 m group (*P* > 0.05; Figures 3B,C). The quantitative data described above are presented in Table 1.

**Table 1:** Concentrations of short-chain fatty acids.

	**Sham-12 m**	**MCAO-1.5 m**	**MCAO-6 m**	**MCAO-12 m**
Acetate (μg/g)	1002.0 ± 69.4	935.1 ± 105.5	645.6 ± 97.8	591.2 ± 106.5
Butyrate (μg/g)	439.5 ± 44.5	353.2 ± 45.8	253.7 ± 20.7	169.3 ± 37.2
Propionate (μg/g)	514.7 ± 39.5	463.6 ± 53.7	292.4 ± 42.5	226.9 ± 44.2

*The data are mean ± SD*.

### Intestinal Histopathological Damage After MCAO

**Figure 4:**
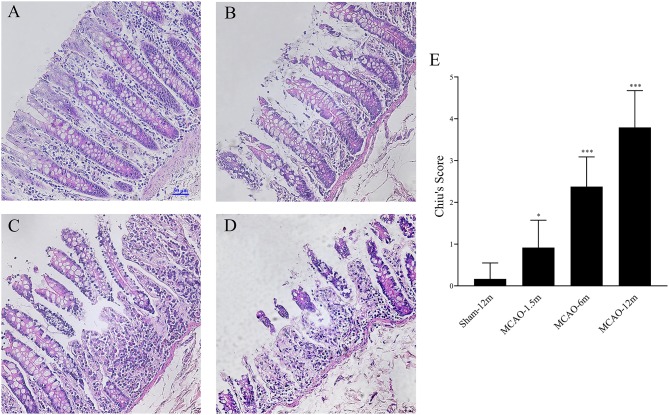
Intestinal histopathological damage after cerebral infarction and evaluation of intestinal injury by Chiu's scores [hematoxylin and eosin (HE) staining, 200×]. **(A)** Representative image from the sham-12 m group. **(B)** Representative image from the MCAO-1.5 m group. **(C)** Representative image from the MCAO-6 m group. **(D)** Representative image from the MCAO-12 m group. **(E)** Changes in the intestinal mucosa Chiu's scores show that the severity of intestinal injury was significantly aggravated after MCAO for 1.5 months (1, 0.25–1), 6 months (2.5, 2–3), and 12 months (4, 3–4.75) compared with that in the sham-12 m group (0, 0–0). The data are presented as the median and interquartile range; *n* = 4 monkeys per group.

Intestinal mucosal damage was observed in all MCAO groups. As shown in Figure 4A, the villi and mucosal epithelia remained intact in the transverse colon of the sham-12 group. An enlarged subepithelial Gruenhagen's space was observed in the MCAO-1.5 m group (Figure 4B). Epithelial lifting down and extension of subepithelial Gruenhagen's space in the transverse colon of the MCAO-6 m group are shown in Figure 4C. In the MCAO-12 m group, the intestinal mucosa showed significant damage, such as denuded villi and disintegration of the lamina propria (Figure 4D). Chiu's score was used to semi-quantitatively investigate intestinal mucosal damage. Chiu's score of the MCAO-1.5 m group (1, 0.25–1) was significantly higher than that of the sham-12 m group (0, 0–0) (*P* < 0.05), as were the scores of the MCAO-6 m (2.5, 2–3) and MCAO-12 m groups (4, 3–4.75) (*P* < 0.001; Figure 4E).

### Intestinal Mucosal Barrier Disruption and Systemic Inflammation

**Figure 5:**
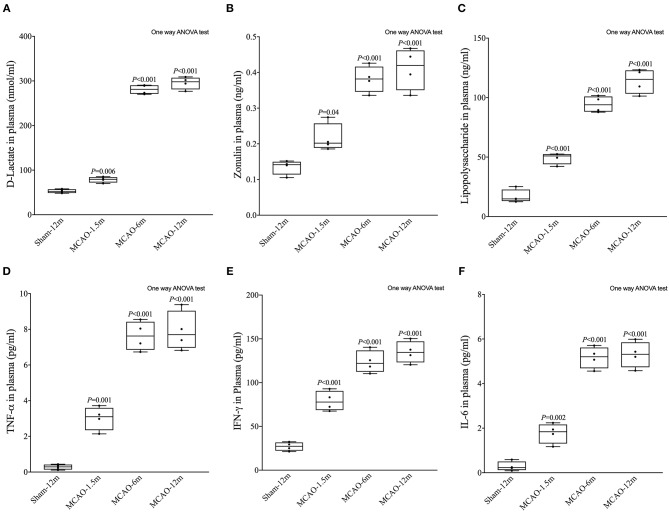
Effects of cerebral infarction on intestinal permeability and the systemic inflammatory response. **(A)** The concentration of D-lactate in plasma was significantly higher in the MCAO-1.5 m (78.63 ± 6.34 nmol/ml), MCAO-6 m (280.8 ± 10.49 nmol/ml), and MCAO-12 m (295.5 ± 13.83 nmol/ml) groups than that in the sham-12 m group (52.69 ± 4.37 nmol/ml) **(B)**. The concentration of zonulin in plasma was significantly higher in the MCAO-1.5 m (0.22 ± 0.040 ng/ml), MCAO-6 m (0.38 ± 0.037 ng/ml), and MCAO-12 m (0.41 ± 0.058 ng/ml) groups than that in the sham-12 m group (0.14 ± 0.020 ng/ml). **(C)** The lipopolysaccharide concentration was significantly higher in the MCAO-1.5 m (49.14 ± 4.86 ng/ml), MCAO-6 m (94.4 ± 6.80 ng/ml), and MCAO-12 m (113.8 ± 10.4 ng/ml) groups than that in the sham-12 m group (113.8 ± 10.4 ng/ml). **(D)** The concentration of TNF-α was significantly increased in the MCAO-1.5 m (3.02 ± 0.66 pg/ml), MCAO-6 m (7.63 ± 0.82 pg/ml), and MCAO-12 m (7.90 ± 1.10 pg/ml) groups compared with that in the sham-12 m group (0.28 ± 0.14 pg/ml). **(E)** The concentration of IFN-γ was significantly higher in the MCAO-1.5 m (79.01 ± 11.36 pg/ml), MCAO-6 m (123.7 ± 12.76 pg/ml), and MCAO-12 m (134.9 ± 12.48 pg/ml) groups than that in the sham-12 m group (27.05 ± 4.87 pg/ml). **(F)** The concentration of IL-6 was significantly higher in the MCAO-1.5 m (1.77 ± 0.45 pg/ml), MCAO-6 m (5.17 ± 0.48 pg/ml), and MCAO-12 m (5.30 ± 0.58 pg/ml) groups than that in the sham-12 m group (0.29 ± 0.21 pg/ml). The data are presented as the mean ± SD; *n* = 4 monkeys per group.

D-lactate, zonulin, and LPS in plasma were detected to evaluate the permeability of the intestinal mucosal barrier. The concentrations of D-lactate (*P* = 0.006 for MCAO-1.5 m vs. sham-12 m; *P* < 0.001 for MCAO-6 m vs. sham-12 m; *P* < 0.001 for MCAO-12 m vs. sham-12 m, Figure 5A), zonulin (*P* = 0.04 for MCAO-1.5 m vs. sham-12 m; *P* < 0.001 for MCAO-6 m vs. sham-12 m; *P* < 0.001 for MCAO-12 m vs. sham-12 m, Figure 5B), and LPS (*P* < 0.001 for MCAO-1.5 m vs. sham-12 m; *P* < 0.001 for MCAO-6 m vs. sham-12 m; *P* < 0.001 for MCAO-12 m vs. sham-12 m, Figure 5C) in plasma were significantly higher in all MCAO groups than those in the sham-12 m group, indicating intestinal mucosal barrier disruption. Furthermore, we detected TNF-α, IFN-γ, and IL-6 in plasma to evaluate the systemic inflammatory response. Compared with the sham-12 m group, the plasma TNF-α (*P* = 0.001 for MCAO-1.5 m vs. sham-12 m; *P* < 0.001 for MCAO-6 m vs. sham-12 m; *P* < 0.001 for MCAO-12 m vs. sham-12 m, Figure 5D), IFN-γ (*P* < 0.001 for MCAO-1.5 m vs. sham-12 m; *P* < 0.001 for MCAO-6 m vs. sham-12 m; *P* < 0.001 for MCAO-12 m vs. sham-12 m, Figure 5E), and IL-6 (*P* = 0.002 for MCAO-1.5 m vs. sham-12 m; *P* < 0.001 for MCAO-6 m vs. sham-12 m; *P* < 0.001 for MCAO-12 m vs. sham-12 m, Figure 5F) levels in all MCAO groups were significantly elevated. The quantitative data described above are presented in Table 2.

**Table 2:** Biomarkers of intestinal mucosal barrier permeability and systemic inflammatory cytokines.

	**Sham-12 m**	**MCAO-1.5 m**	**MCAO-6 m**	**MCAO-12m**
D-Lactate (nmol/ml)	52.69 ± 4.37	78.63 ± 6.34	280.8 ± 10.49	295.5 ± 13.83
Zonulin (ng/ml)	0.14 ± 0.020	0.22 ± 0.040	0.38 ± 0.037	0.41 ± 0.058
LPS (ng/ml)	16.85 ± 5.70	49.14 ± 4.86	94.4 ± 6.80	113.8 ± 10.4
TNF-α (pg/ml)	0.28 ± 0.14	3.02 ± 0.66	7.63 ± 0.82	7.90 ± 1.10
IFN-γ (pg/ml)	27.05 ± 4.87	79.01 ± 11.36	123.7 ± 12.76	134.9 ± 12.48
IL-6 (pg/ml)	0.29 ± 0.21	1.77 ± 0.45	5.17 ± 0.48	5.30 ± 0.58

*The data are mean ± SD*.

### The Bacteroidetes Level Positively Correlated With Plasma LPS and Inflammatory Cytokines After MCAO

**Figure 6:**
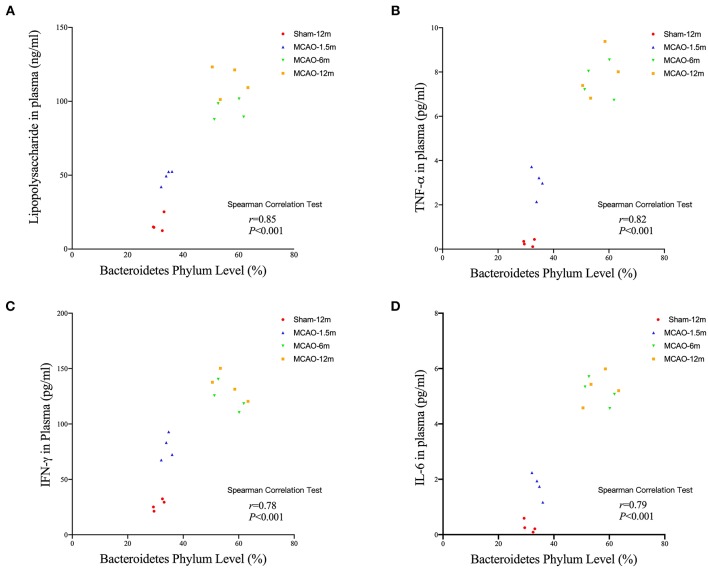
Correlation analysis between the Bacteroidetes level, plasma lipopolysaccharide (LPS), and inflammatory cytokines in cynomolgus monkeys after the MCAO procedure. The relative Bacteroidetes level has significant positive correlations with **(A)** LPS (*r* = 0.85, *P* < 0.001), **(B)** tumor necrosis factor (TNF)-α (*r* = 0.82, *P* < 0.001), **(C)** interferon (IFN)-γ (*r* = 0.78, *P* < 0.001), and **(D)** interleukin (IL)-6 (*r* = 0.79, *P* < 0.001).

The Spearman correlation analysis showed that the relative abundance of Bacteroidetes level had significant positive correlation with LPS (*r* = 0.85, *P* < 0.001, Figure 6A), TNF-α (*r* = 0.82, *P* < 0.001, Figure 6B), IFN-γ (*r* = 0.79, *P* < 0.001, Figure 6C), and IFN-γ (*r* = 0.78, *P* < 0.001, Figure 6D).

## Discussion

In this study, we found gut microbiota dysbiosis with elevation of the Bacteroidetes phylum, along with increasing levels of the *Prevotella* genus and decreasing levels of the Firmicutes phylum and *Faecalibacterium, Streptococcus, Lactobacillus*, and *Oscillospira* genera after cerebral infarction in cynomolgus monkeys. SCFAs, which are important immunomodulators produced by gut bacteria fermentation that can suppress the LPS-induced inflammatory response (32) and decrease production of proinflammatory cytokines (14), were decreased 6 and 12 months after cerebral infarction. In addition, intestinal mucosal damage, as well as plasma LPS levels and inflammatory cytokines, continued to increase after cerebral infarction. The current findings suggest that gut microbiota dysbiosis along with intestinal mucosal damage and chronic systemic inflammation persist after cerebral infarction. The damaged intestinal mucosal barrier and variations in the microbiota composition potentially disrupt systemic immune homeostasis.

Although evidence has shown that the gut microbiota affect stroke outcome in rodents (3, 4), supporting the concept of bidirectional communication along the brain–gut–microbiota axis, no reports have examined long-term changes in the gut microbiota and effects on the host. Using 16S rDNA sequencing, we confirmed an increase in Bacteroidetes at the phylum level after cerebral infarction. An elevated Bacteroidetes phylum abundance was also found 3 days after ischemic stroke onset in mice and is considered a hallmark of poststroke dysbiosis (4). Additionally, in that murine study, decreased alpha diversity was shown, which indicated decreased species richness after ischemic stroke (4). However, a clinical research study, in which fecal samples were collected within 48 h after admission, showed decreased Bacteroidetes levels and increased alpha diversity of the microbiota in acute ischemic stroke and transient ischemic attack patients (7). The results from murine and human studies are therefore inconsistent. Stroke patients' daily diet as well as comorbidities such as hypertension, diabetes, and obesity may influence the gut microbiota (8–11). In addition, the anatomical structures and behavior of rodents significantly differ from those of humans and may influence the gut microbiota. In our study, we did not find a significant difference in the alpha diversity after cerebral infarction for 1 year in cynomolgus monkeys. Evidence shows that the gut microbiome of cynomolgus monkeys exhibits strong similarity to the human gut microbiome (13). Our findings may provide an experimental basis for future preclinical studies of the interaction between the microbiota and stroke.

*Prevotella*, which belongs to the Bacteroidetes phylum, plays an important proinflammatory role in chronic inflammatory diseases in humans (33). In our study, we identified an increased relative abundance of Prevotella in MCAO-operated monkeys, which suggests that this genus may be associated with the poststroke inflammatory response. Decreased relative abundance levels of *Faecalibacterium, Streptococcus, Lactobacillus*, and *Oscillospira* genera, which belong to the Firmicutes phylum, were observed in MCAO-operated monkeys in our study. The *Faecalibacterium* and *Oscillospira* genera have been widely recognized as a major source of butyrate in the host (34, 35). Butyrate, the most potent SCFA, plays a critical role in maintaining the integrity of the intestinal epithelial barrier and inhibiting proinflammatory cytokine production and is also considered a therapeutic target for brain disorders (36). In our study, we observed a decrease in the concentration of butyrate in the plasma of the MCAO-operated monkeys when compared to the sham-12 m group, which may be linked to decreases in *Faecalibacterium* and *Oscillospira*. The decreased plasma SCFA levels observed in our study at 6 and 12 months after the MCAO operation showed that chronic gut microbiota dysbiosis may also hinder the production of SCFAs.

*Lactobacillus* and *Streptococcus* were also the major gut microbiome communities in all groups. *Lactobacillus* is an important genus of probiotic bacteria for the host, and its relative abundance level decreased after cerebral infarction in our study. Supplementation with *Lactobacillus* has been proven to enhance cognitive function, improve mood, and attenuate aging-associated inflammation (37–39). Poststroke dementia and depression are common complications in survivors of stroke (40, 41), and we identified chronic systemic inflammation after cerebral infarction in cynomolgus monkey. Whether *Lactobacillus* supplementation is beneficial to stroke patients remains unclear and needs to be investigated in future studies. The relative abundance level of *Streptococcus* was also decreased in our study. *Streptococcus* genus includes both probiotic bacteria (such as *Streptococcus thermophilus*) (42) and pathogenic bacteria (such as *Streptococcus pneumoniae*) (43). However, in our study, 16S rDNA sequencing, which was used to evaluate the abundance and composition of gut microbiota, identified gut microbiota from phylum to genus level but not to species level. Thus, the exact roles of intestinal *Streptococcus* in cerebral infarction remains to be explored in future studies.

Increased LPS concentrations in blood have been reported to induce systemic inflammation and impair stroke outcome (44). These data indicate that gut microbiota dysbiosis may be an important contributing factor for poststroke complications. Because we observed an increased relative abundance of Bacteroidetes, a phylum of gram-negative bacteria (44), in MCAO-operated monkeys, we further investigated whether plasma LPS levels were elevated. We observed a significant increase in LPS levels in the plasma of MCAO-operated monkeys, especially 6 and 12 months after MCAO. Thus, LPS may play an important role in poststroke chronic systemic inflammation. Notably, we observed intestinal mucosal barrier disruption and morphological damage to the intestinal mucosa in the MCAO-operated monkeys. The damaged intestinal mucosal barrier may be associated with increased release of LPS from the gut to the blood. Furthermore, we confirmed that the proinflammatory cytokines IFN-γ, TNF-α, and IL-6 were elevated in plasma even 12 months after MCAO compared with those in the sham-12 m group, indicating that chronic systemic inflammation persists after stroke onset. These findings suggest that not only gut microbiota dysbiosis but also chronic systemic inflammation persists after cerebral infarction. Correlation analysis also revealed that the increased plasma LPS or inflammatory cytokine levels and Bacteroidetes overgrowth were closely related. On the other hand, the immune system, gut histology, and permeability may be affected by external factors such as diet, infection, and housing sanitation. Thus, in our study, we controlled these factors for all animals to minimize the changes in the gut microbiota, gut histology, and cytokines induced by these factors. Furthermore, no significant signs of infection (e.g., cough, fever, suppuration, or diarrhea) were observed in any monkeys during our study.

Our study confirmed the chronic systemic inflammatory response in an NHP stroke model and the persistence of poststroke gut microbiota dysbiosis. We may infer that the poststroke chronic systemic inflammatory response may impact the central nervous system as the chronic systemic inflammatory response has been proven to be associated with cognitive impairment, deficits in learning and memory, and depression and anxiety (38). Proinflammatory cytokines released in the periphery are involved in peripheral immune system-to-brain communication (38). Thus, the poststroke gut microbiota and chronic systemic inflammation may be therapeutic targets for improving stroke outcomes.

However, our study also has limitations. First, we demonstrated the persistence of gut microbiota dysbiosis and the chronic systemic inflammatory response after cerebral infarction, but we did not identify their effects on stroke outcomes. Further studies will be performed to confirm the bidirectional association between them. Second, to prevent post-operative infection, all monkeys were injected with penicillin once a day for 2 days, potentially inducing gut microbiota dysbiosis. We found that short-term usage of penicillin had no significant long-term influence on the gut microbiota in our study. However, we only assessed gut microbiota 1.5 months after intravenous injection of the agents. Hence, changes on earlier time points cannot be excluded. Third, we only collected samples from the sham group 12 months after surgery, which is a limiting factor of our study. We controlled the diet, sanitation levels, the housing environment, antibiotic use, and infection to minimize the effects of external factors on the gut microbiota. In addition, significant differences in the gut microbiota, SCFAs levels, intestinal mucosal damage, and plasma cytokine concentrations were found between the MCAO-12 m and sham-12 m groups, which may reflect the long-term impact of stroke on the gut microbiota and systemic inflammation.

In conclusion, our study provides evidence for long-term gut microbiota dysbiosis in cynomolgus monkeys, accompanied by perturbations in SCFAs levels, intestinal mucosal damage, and increased plasma inflammatory cytokine concentrations as compared to the sham-12 m group, which may reflect the long-term impact of stroke on the gut microbiota and systemic inflammation.

## Data Availability

The raw data supporting the conclusions of this manuscript will be made available by the authors, without undue reservation, to any qualified researcher.

## Ethics Statement

The study was approved by the Institutional Animal Care and Use Committee of Guangdong Landau Biotechnology Co., Ltd.

## Author Contributions

YC, JhL, and JZ designed the study. YC, JhL, FO, XC, and YL performed the experiments. YC and JhL analyzed data and wrote the initial draft. TL, ZJ, and JnL provided advice in study design and execution. JZ reviewed the results. All authors contributed to the final draft and agreed to submit the manuscript for publication.

### Conflict of Interest Statement

YL was employed by Guangdong Landau Biotechnology Co., Ltd., Guangzhou, China. The remaining authors declare that the research was conducted in the absence of any commercial or financial relationships that could be construed as a potential conflict of interest.
