# Supplementary figures and images for: Persistence of Gut Microbiota Dysbiosis and Chronic Systemic Inflammation After Cerebral Infarction in Cynomolgus Monkeys
Source: Front Neurol. 2019 Jun 28;10:661. doi: 10.3389/fneur.2019.00661 (PMC6611357; doi:10.3389/fneur.2019.00661)

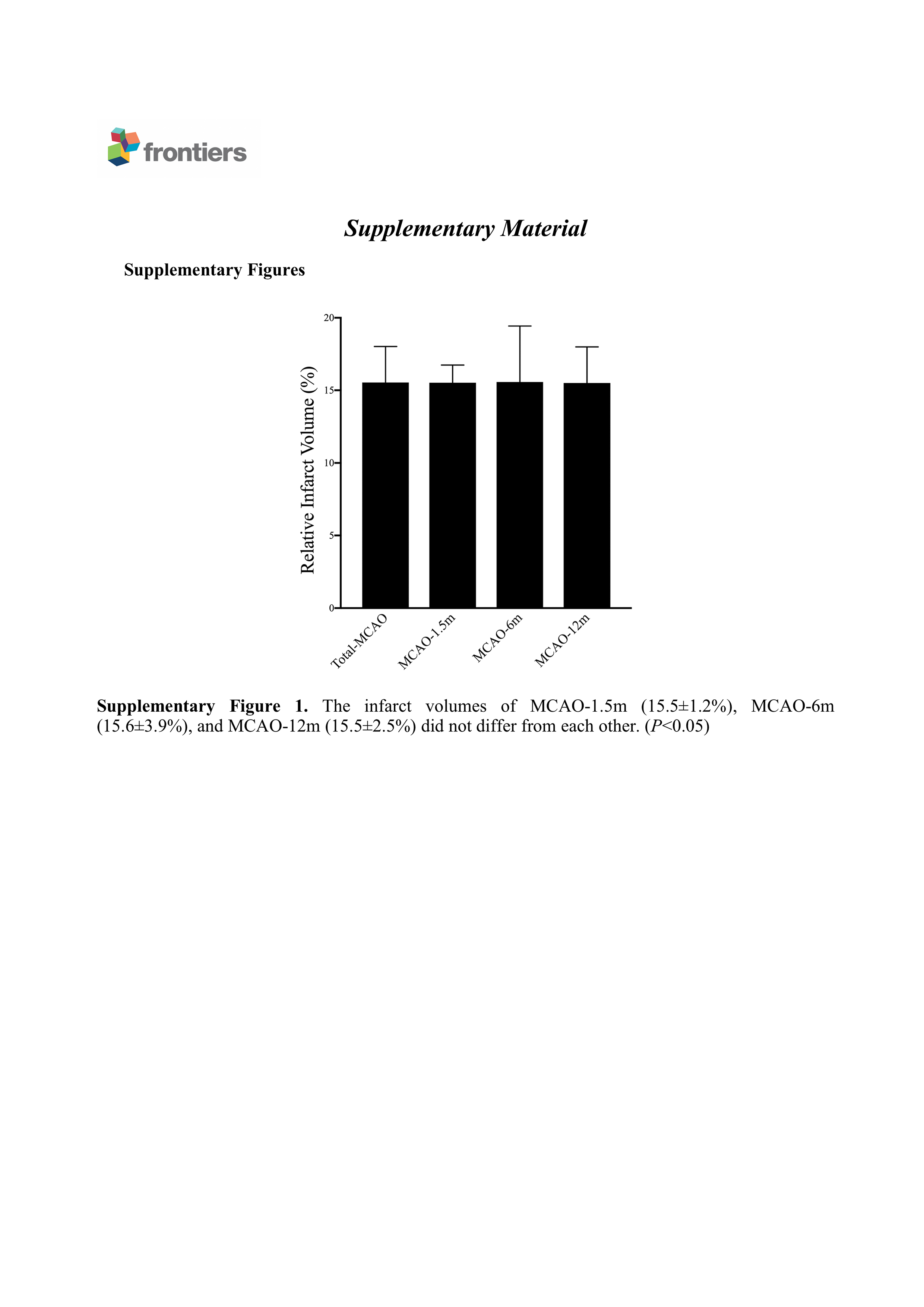

Supplement: Supplementary file 1 [file Image_1.TIF]

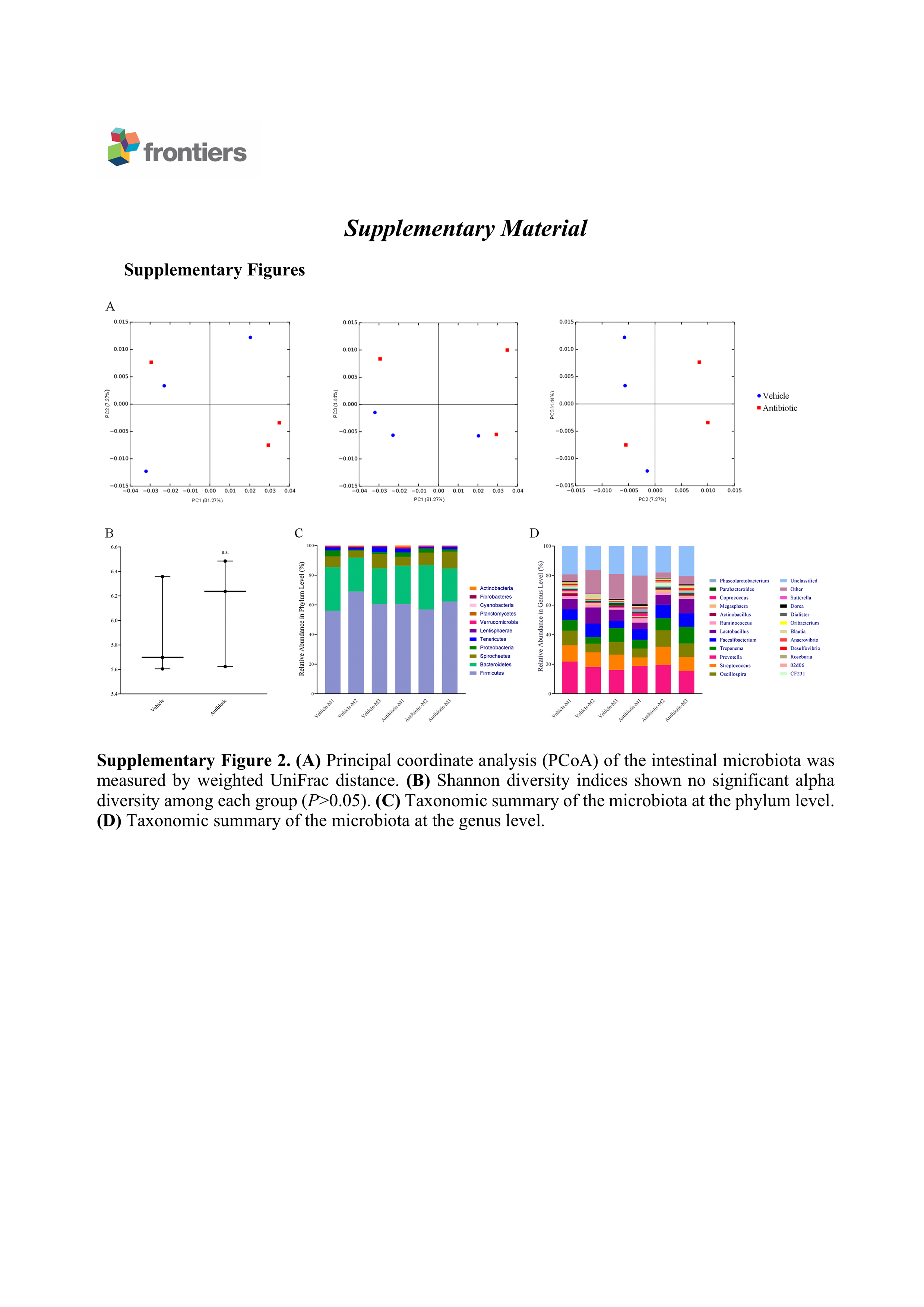

Supplement: Supplementary file 2 [file Image_2.TIF]

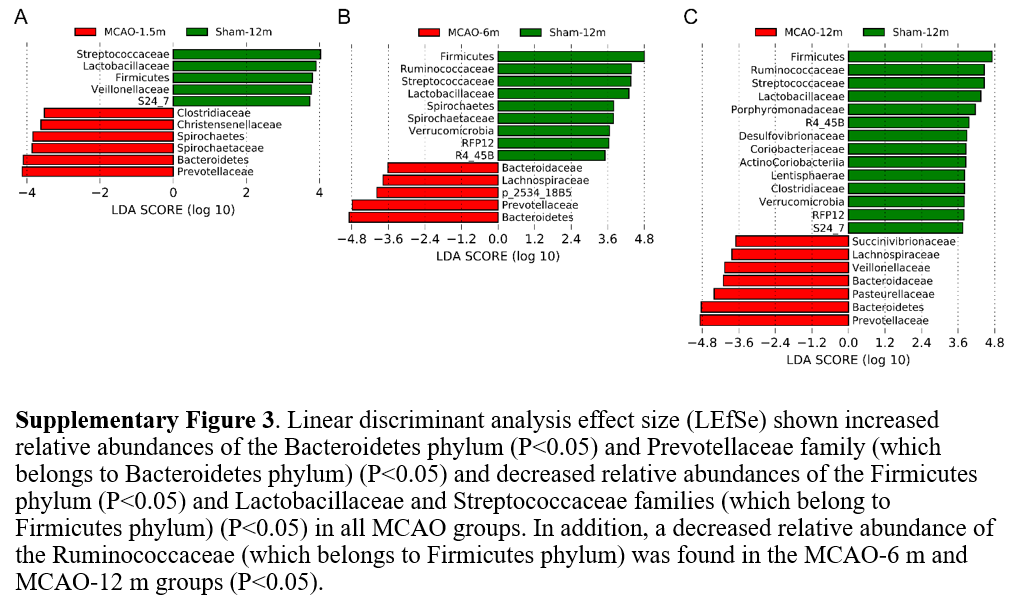

Supplement: Supplementary file 3 [file Image_3.TIF]
